# Supplementary material for: MetaRibo-Seq measures translation in microbiomes
Source: Nat Commun. 2020 Jun 29;11:3268. doi: 10.1038/s41467-020-17081-z (PMC7324362; doi:10.1038/s41467-020-17081-z)
Supplement: Supplementary file 10 — Supplementary Data 7 [file 41467_2020_17081_MOESM10_ESM.zip › File2/Confidence_High_Taxonomy/387168_out.krona.html]

Javascript must be enabled to view this page.

members
magnitude
magnitudeUnassigned
count
unassigned
taxon
rank

387168\_out

9

superkingdom
2
9

9
phylum
976

9
class
200643

171549
order
9

family
815
9

816
genus
9


SRS022713\_contig\_number\_2183
818
species
1

671267
species
2

SRS024388\_contig\_number\_3200SRS1041132\_contig\_number\_6147

6
species
821

SRS013158\_contig\_number\_5234SRS015960\_contig\_number\_16091SRS020328\_contig\_number\_35073SRS023971\_contig\_number\_3415SRS057478\_contig\_number\_4932SRS064973\_contig\_number\_379
